# A smart switching system to enable automatic tuning and detuning of metamaterial resonators in MRI scans

**DOI:** 10.1038/s41598-020-66884-z

**Published:** 2020-06-22

**Authors:** Shimul Saha, Roberto Pricci, Maria Koutsoupidou, Helena Cano-Garcia, Ditjon Katana, Srinivas Rana, Panagiotis Kosmas, George Palikaras, Andrew Webb, Efthymios Kallos

**Affiliations:** 1 MediWiSe| Medical Wireless Sensing Ltd, Queen Mary Bio Enterprise Innovation Centre, 42 New Road, E1 2AX London, UK; 2 Metamaterial Inc, 1 Research Drive, Dartmouth, Nova Scotia B2Y 4M9 Canada; 3 Department of Engineering, King’s College London, London, WC2R 2LS UK; 4 C.J. Gorter High Field Magnetic Resonance Center, Leiden University Medical Center, Albinusdreef 2, 2333 ZA Leiden, The Netherlands

**Keywords:** Electrical and electronic engineering, Magnetic resonance imaging

## Abstract

We present a radio-frequency-activated switching system that can automatically detune a metamaterial resonator to enhance magnetic resonance imaging (MRI) performance. Local sensitivity-enhancing metamaterials typically consist of resonant components, which means that the transmitted radio frequency field is spatially inhomogeneous. The switching system shows for the first time that a metamaterial resonator can be detuned during transmission and tuned during reception using a digital circuit. This allows a resonating system to maintain homogeneous transmit field while maintaining an increased receive sensitivity. As a result, sensitivity can be enhanced without changing the system-provided specific absorption rate (SAR) models. The developed digital circuit consists of inductors sensitive to the transmit radio-frequency pulses, along with diodes acting as switches to control the resonance frequency of the resonator. We first test the automatic resonator detuning on-the-bench, and subsequently evaluate it in a 1.5 T MRI scanner using tissue-mimicking phantoms. The scan results demonstrate that the switching mechanism automatically detunes the resonator in transmit mode, while retaining its sensitivity-enhancing properties (tuned to the Larmor frequency) in receive mode. Since it does not require any connection to the MRI console, the switching system can have broad applications and could be adapted for use with other types of MRI scanners and field-enhancing resonators.

## Introduction

Magnetic Resonance Imaging (MRI) is one of the most commonly used clinical diagnostic scanning techniques^1–3^. In general, higher static magnetic fields increase the signal-to-noise ratio (SNR), thereby leading to higher spatial resolution images^4,5^. The SNR at lower magnetic fields can also be increased by improving the sensitivity of the receiving coils used in the MRI scanner^6,7^. Typically, a large number (8 to 64) of receiver coils are placed close to the imaging region-of-interest for maximum sensitivity. High-power radio-frequency (RF) pulses are transmitted by transmit coils, typically embedded in the bore of the scanner, to produce coherent processing magnetization in the patient. The return RF pulses are then collected by the receive coils. These are detuned during the transmit sequence to prevent unwanted induced currents^8^. Similarly, the transmit coils are detuned during the receive sequence to reduce image artefacts and signal loss^9,10^.

Obtaining higher SNR via higher static magnetic field strength has drawbacks, such as increased tissue heating, nausea, and challenges in maintaing uniform fields over large regions^11–13^. For that reason, alternative methods based on metamaterial structures^14–18^, have been proposed to improve the sensitivity of MRI scans without increasing the static field strength. These include split ring resonators^19^, swiss rolls^20^, curved wire mediums^21^ and spiral shape coils^22^, as well as metamaterial resonators previously reported by the authors^23,24^. The benefit of such structures is that they support resonance modes that enhance the local RF magnetic field of the scanner in the region of interest, and thus increase the scanner sensitivity.

However, since these resonant structures are tuned to the Larmor frequency during both the transmit and receive sequences, they have mitigating disadvantages. First, they cause the transmitted field to become very inhomogeneous near the patient. Unlike the field inhomogeneities of the receive coils, which can be corrected using image processing algorithms^25^, inhomogeneities in the transmitted RF fields cause spatially-variant tissue contrast which cannot be corrected. A second, more practical problem is that the specific absorption rate (SAR) models of the MRI scanners used to ensure safe scans, can no longer be used.

**Figure 1:**
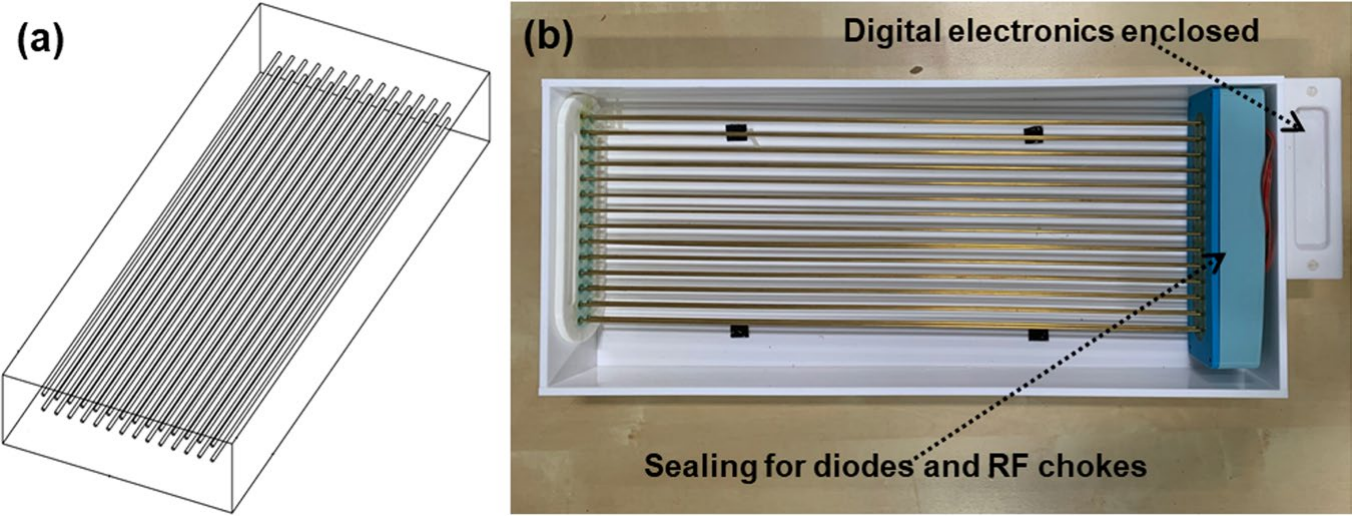
Schematic and assembled resonator. (**a**) A schematic of the metamaterial resonator with two rows of 14 wires embedded in a high dielectric constant medium, deionised (DI) water^23,24^. (**b**) The resonator with the switching circuit is placed inside an acrylic box (440 mm × 180 mm × 50 mm). The box is filled with DI water to reduce the effective wavelength and operate as a Fabry Perot resonator at 64 MHz. The switching elements are contained in a watertight casing connected to the wires. The rest of the digital circuit and a pickup inductor for generating the switching signal (clock) is housed inside a case on the outside.

Therefore, it is highly desirable to design a detunable metamaterial resonator which can maintain homogeneous excitation in the transmit mode, without sacrificing the increased receiver sensitivity. This is the focus of this work, where we achieve this by employing an active switching system that automatically detunes a metamaterial resonator consisting of an array of parallel metallic wires (Fig. 1) during the transmit phase, while it tunes it to the Larmor frequency of 64 MHz at 1.5 T during the receive phase. This is achieved without a physical connection to the MRI scanner. Detuning the resonator in transmit phase, enable scan to be performed safely using MRI scanner provided SAR models.

The core element of the switching system is an array of diodes. The diodes act as switching elements, mounted between every pair of adjacent wires of the resonator. These diodes are activated by a sensing digital circuit, which picks up the energy of the transmitted RF magnetic (B_1_^+^) field through an inductor and generates a low frequency square wave that turns on the switches, thus shorting each pair of adjacent wires causing them to have a longer electrical length. This results in detuning, as the resonator’s frequency is shifted down far away from the Larmor frequency. In the receive phase, when no B_1_^+^ field is present, the switches remain off and the metamaterial resonates at the Larmor frequency. Overall, this means that the local receive sensitivity is enhanced, but the transmitted field remains unaffected and spatially homogeneous.

While a nonlinear metamaterial helical array has been shown to provide selective tuning based on a voltage-dependent capacitance of a varactor diode in^26^, this work presents a switching system which relies solely on digital circuits and thus can be applied to any type of resonating structure aimed at enhancing MRI scans.

The paper first presents the methodology to control the resonator’s frequency in a laboratory environment using manual push button switches. A digital sensing circuit is then integrated and tested on the bench. Finally, the same system is tested in a 1.5 T MRI scanner by imaging tissue-mimicking phantoms placed on top of the metamaterial resonator.

## Methods

### Slider switches for manual tuning and detuning

**Figure 2:**
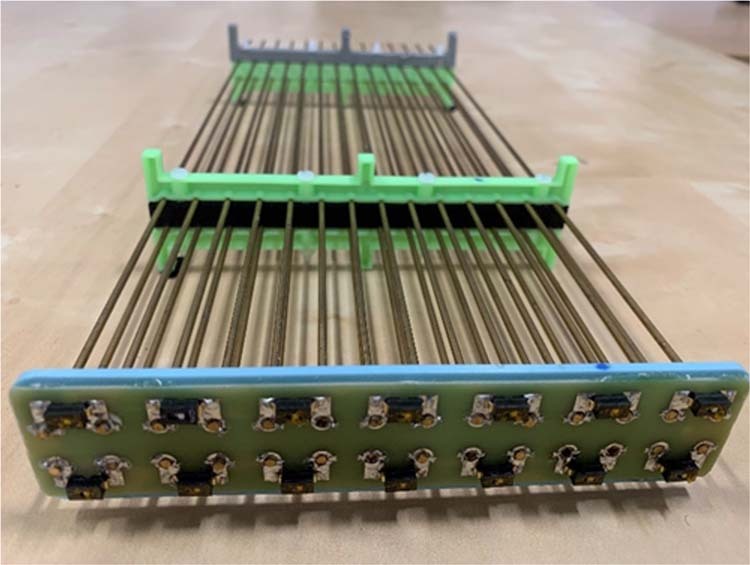
The assembled wire matrix of the metamaterial with DIP switches connected between each pair of wires. The plastic holder and spacer were used to keep the wires in parallel and equidistant. A printed circuit board was used for mechanical stability and soldering the switches onto the pads.

The metamaterial resonator comprises an array of 2 × 14 brass wires of 2 mm diameter. The length of each wire is 372 mm, which is tuned to resonate at the Larmor frequency in water. The wires are 10 mm apart from each other both horizontally and vertically. We have used 3D printed plastic spacers to keep these wires apart and parallel to each other (Fig. 2). We placed the assembly in an acrylic box (422 mm × 180 mm × 50 mm) filled with DI water.

Electromagnetic simulations have shown that a meaningful resonance frequency shifts occurs when the wires are connected horizontally (adjacent wires within the same row). As an early proof of concept, we can observe the shift by manually shorting adjacent wires. We soldered surface mount dual in-line package (DIP) switches (Omron, UK) on a PCB connecting the ends of two horizontally adjacent wires to a switch pad through a 3-way vertical PCB socket (Fig. 2). The switches can be turned on and off by sliding a small button which in turn shorts and opens the associated pair of wires. Finally, we used a non-resonant loop antenna on top of the acrylic box to measure the frequency characteristics of the metamaterial using a Keysight E8361A vector network analyser (VNA).

### Semiconductor diodes for electronic and automatic switching

**Figure 3:**
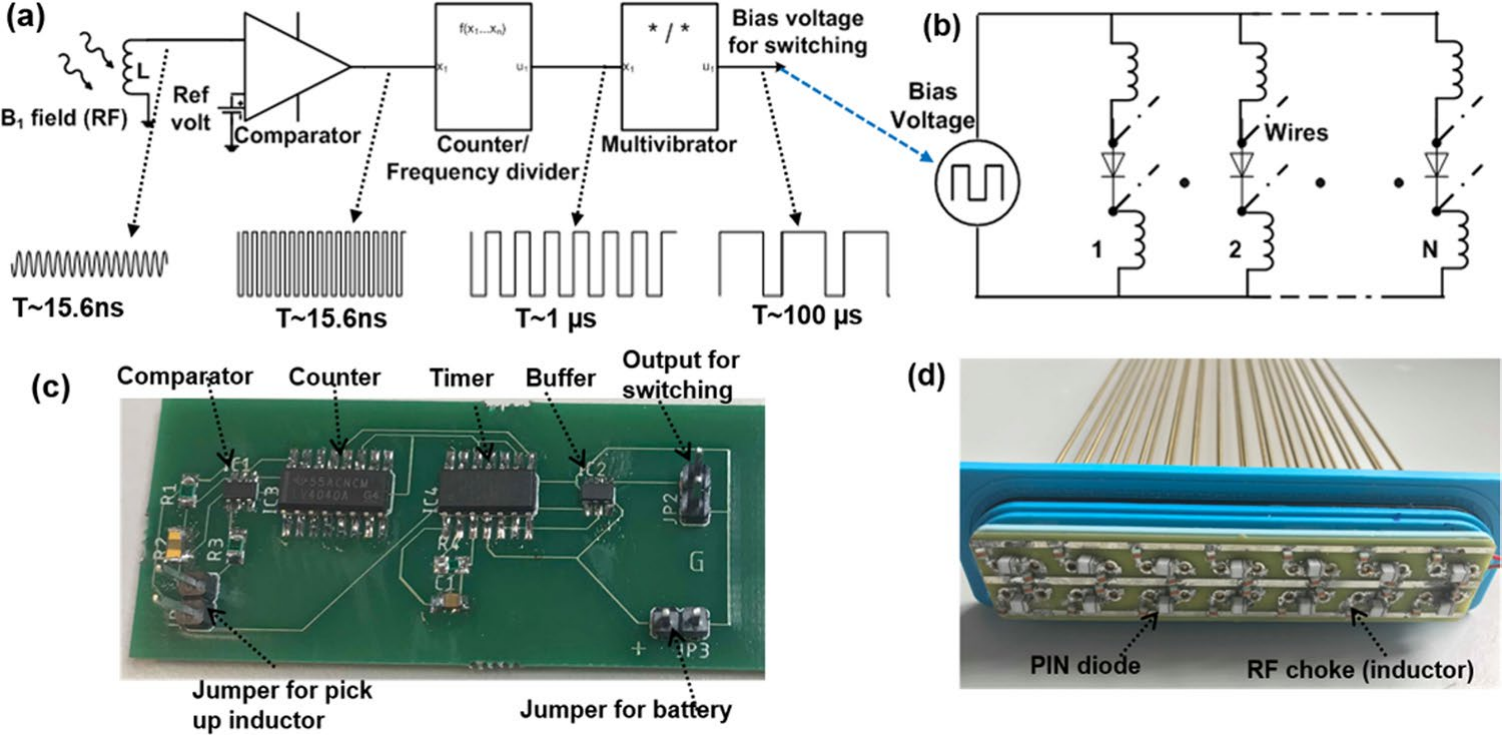
The digital sensor design and assembly. (**a**) The block diagram of the digital circuit to detect the B_1_/RF field via an inductor, and convert it to a rail to rail square wave, which further down converted from 64 MHz to 10 kHz via an asynchronous counter and monostable multivibrator with duty cycle of >90%. A representative wave diagram is shown at each point of the circuit when an RF pulse is picked up by the inductor during transmission sequence (**b**) The circuit diagram of the switching matrix connected to the parallel wires of the metamaterial.(**c**) The respective assembled PCB (The sensing inductor and battery are not shown in the picture) for digital circuits and (**d**) The implemented PCB for switch matrix with a common anode and cathode bias line. RF Choke Inductors are connected to the anode and cathode of each diode to isolate them from each other at higher frequency (64 MHz).

The previous setup allowed the validation of our simulation results. However, an electronic actuation of the switches is preferable in practice. To this end, we used semiconductor switches for the electronic tuning function. The switches are activated by either a clock pulse or a DC signal. These signals can be generated by a control circuit for automatic switching during the MRI scan. High frequency MRI compatible PIN diodes (Macom, USA, MA4P7441F-1091T) with maximum forward bias voltage ~1 V were used as switching elements. The anode and cathode of each diode were connected to each pair of wires (Fig. 3). A pair of chokes (3.3 µH inductors) were used with each diode to isolate them from each other at 64 MHz. A bias voltage (clock) is applied between the anode and cathode with higher potential applied at the anode. All 14 diodes are connected in parallel to the bias line allowing their activation with a single clock or DC source (Fig. 3). A sealed compartment was used to protect the electronics from water (Fig. 1). The metamaterial resonator wires are connected to the diodes with a 3-way vertical PCB socket. The whole device was placed in an acrylic box (440 mm × 180 mm × 50 mm) (Fig. 1), filled with DI water.

### Automatic generation of the detuning control signal using digital electronics

Since the resonator operates without any connection to the MRI console and power system, an automatic mechanism for activating the switch matrix is required. To this end, we have designed and built a digital circuit (Fig. 3a,c respectively) that detects the transmitted RF field and generates a rail to rail square pulse to activate the switches (Fig. 3b,d). A small inductor (3.3 µH) picks up the B_1_ field at 64 MHz during the transmit sequence and it induces a voltage on the order of tens of mV. The voltage output is digitized by a high-speed comparator (LMH7220MK, Texas Instrument, USA). A small potential is used as reference (~20 mV).

When the induced voltage at the inductor is greater than the reference, the comparator generates a R-R clock at the MRI Larmor frequency, i.e. 64 MHz for a 1.5 T scanner. When there is no B_1_ field, the output of the comparator is zero. The output is fed to the clock input of a 12-bit edge triggered asynchronous counter (SN74LV4040AD) to down convert the frequency, e.g. its 8^th^ bit output is 1 MHz. The output of the counter is fed to a timer circuit, a monostable multivibrator (HEF4047BT) which down converts it further to a fixed frequency of approximately 10 kHz, as defined by a RC time constant, with a duty cycle greater than 90%. The frequency and the duty cycle can be adjusted to any desired value by selecting the appropriate counter bit and *RC* values. The output is shaped by a dual inverter (buffer) and fed to the switching electronics.

We have adjusted the clock frequency to around 10 kHz (<100 µs), which is significantly faster compared to typical transmit and receive sequences of 1–5 ms^27^. The frequency can be adjusted with appropriate *RC* elements in the timer circuits. Although the current circuit is configured to generate switching voltage in the MRI transmit mode, it can be easily configured for switching in the receive mode, by either using a multiplexer or an additional inverter logic. The digital circuit is powered by a 3.7 V non-magnetic lithium polymer battery (PGEB-NM651825).

### Bench testing of auto-tuning mechanism

**Figure 4:**
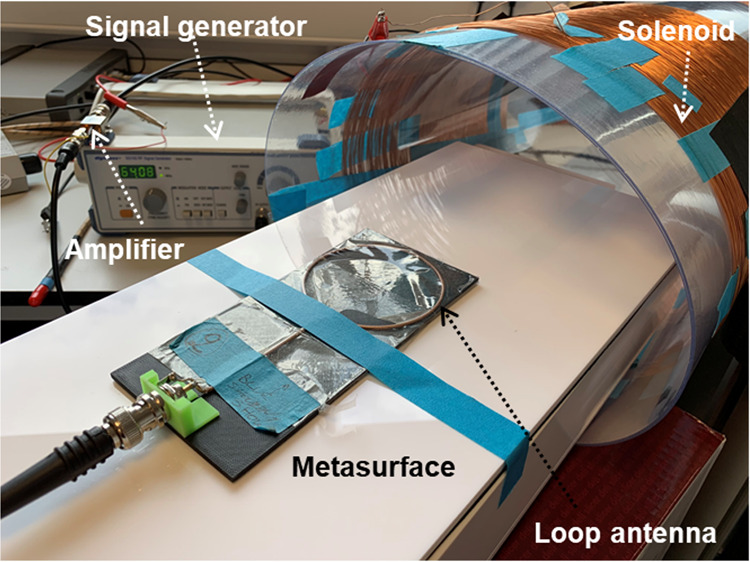
The solenoid made from insulated copper wire to mimic the MRI RF magnetic field for automatic detection. The solenoid is fed by an RF signal generator followed by an 18 dB gain amplifier. A broad band loop coil is used to measure the resonance frequency^23,24^.

We tested the basic circuit functionality in a laboratory environment before evaluating it in a clinical environment with an MRI scanner. We built a solenoid to generate the RF magnetic field distribution similar to that of an MRI scanner during the transmit sequence. The solenoid was made from AWG2 insulated copper wire wrapped 450 times around a 25 cm diameter acrylic tube (Fig. 4). The real part of the impedance is close to 50 Ω for maximizing the power transfer. We excited the solenoid with a Digimess signal generator working at 10 kHz-150 MHz with 50 Ω port impedance. We used an 18-dB gain amplifier from Mini-circuits (ZX60-43-S+) to amplify the signal from the signal generator for feeding the solenoid. For the resonance frequency measurements, we placed the digital circuit with the pick-up inductor at the centre of the solenoid and turned the RF source on and off manually to generate the RF/H field. The inductor generates a small voltage by picking up the RF field and the digital circuit converts it to a low frequency clock as described above. We used the output of the digital circuit to activate the switches shown in Fig. 3d and we measured the resonance frequency with a loop coil during the experiment.

### Evaluation of switch circuits in a 1.5 T MRI

Prior to measurements in an MRI scanner, we tested for safety purposes the digital electronics and associated components with a magnetically induced displacement force test by MR: Comp, Germany. The deflection of the test objects (the electronics) were qualitatively observed to be less than 45° - the limit of deflection, which means that the displacement force exerted on the test objects is smaller than the force of gravity and therefore safe to operate in an MRI of a particular strength (1.5 T for our case)^28^.

**Figure 5:**
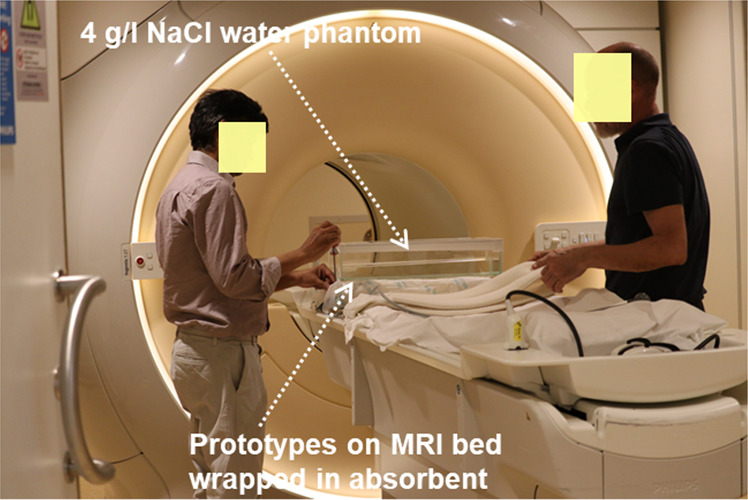
Experimental setup in the 1.5 T MRI scanner. The metamaterial prototype placed on the MRI scanner’s table. The prototype was wrapped with an absorbent mat to prevent water leakages. A 4 g/L NaCl solution was used as a human body mimicking phantom for the scan to mimic the body conductivity.

To evaluate the auto-tuning mechanism of the switch matrix, we used the resonator for scanning a water phantom in a Philips Ingenia 1.5 T MRI system at C. J Gorter Center for High Field MRI, Leiden, The Netherlands (Fig. 5). To mimic the human body conductivity, we used a 4 g/L salt solution as phantom prepared for the scan^29^. The phantom was scanned by placing it on top of the metamaterial (Fig. 5). We used a low flip angle (nominal 10 degrees with metamaterial detuned) T_1_ weighted gradient echo sequence for a transverse scan with following parameters: TR 9.5 ms, TE 4.8 ms, data matrix 672 ×672 ×40, spatial resolution 0.96 mm ×0.96 mm ×2 mm, and acquisition time 2 minute 40 seconds.

## Results and discussion

### Manual resonator detuning using slider switches

**Figure 6:**
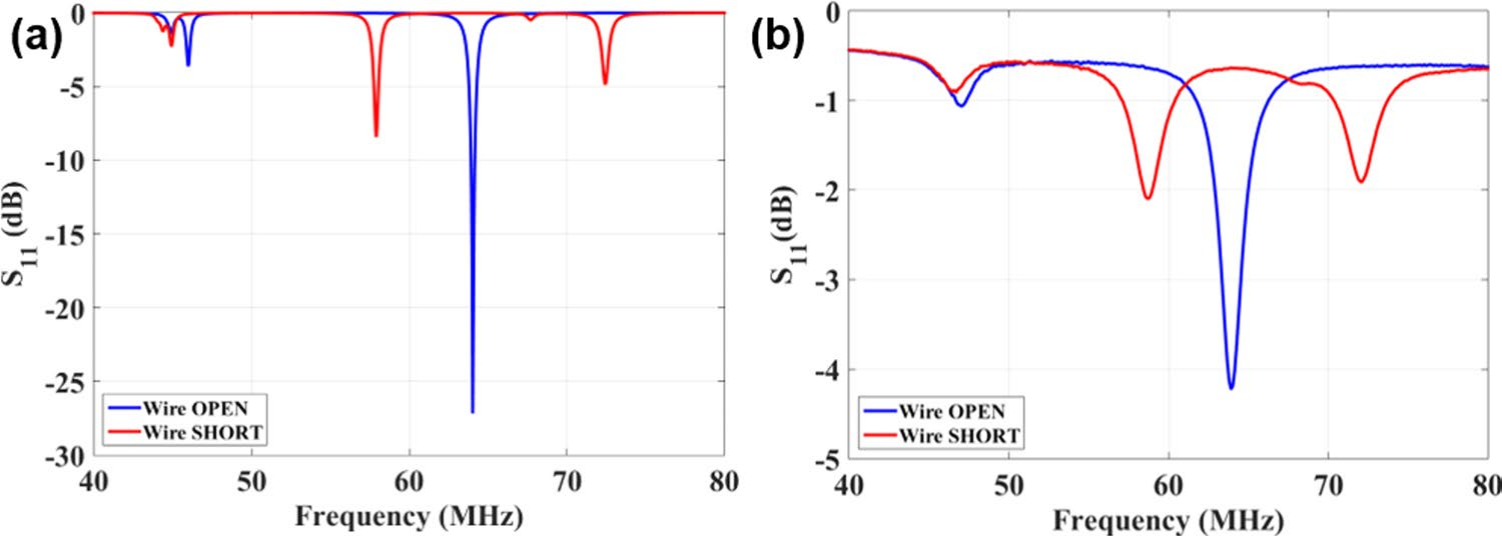
Simulated and measured results. (**a**) Simulation results of the reflection coefficient (S11) of an untuned pickup loop loosely coupled to the metamaterial resonator when the wires are open (blue), and shorted horizontally (red). (**b**) Measured resonance frequency of the metamaterial for the “on” and “off” state of the DIP switches connected horizontally. A frequency shift of ≥5.0 MHz is obtained when the switches are turned on manually.

To identify the best topology of the switches to maximize the resonating frequency detuning, the device was first simulated in three distinct setups: a) unconnected wires, b) pairs of wires shorted horizontally (i.e. electrical contact within the same layer), and c) pairs of wires shorted vertically (i.e. electrical contact between different layers). Figure 6(a) shows the simulated results of the frequency response of the system. A frequency shift of 6.0 MHz was observed when the wires were connected horizontally while the vertical contact did not produce a significant frequency shift (data not shown). The measured frequency response of the system for both the “off” state (wires open; tuned) and the “on” state (wires shorted; detuned) of the switch matrix are shown in Fig. 6(b). Turning the switches to their “on” state shifts the resonance frequency from 64.0 MHz generating two new resonances at 59.0 MHz and 72.0 MHz. The switching provides a minimum shift of 5.0 MHz from the desired frequency while there is no resonance at 64.0 MHz, which closely matches the simulation results. The difference in the frequency shift between simulation and measurements could be attributed to finite resistance (R_short_ ≠ 0 & R_open_ ≠ ∝) of the switch for on and off state. The finite resistance also reduces the Q factor of the device thus broadening its resonance profile.

### Electronic resonator detuning using diode switches

As a next step, we explored electronic switching for resonator detuning. An identical metamaterial was used with 14 diodes (Macom, USA, MA4P7441F-1091T) as switching elements (note that MOSFETs could also potentially be used). We placed pairs of inductors at the anode and cathode of each diode (see Methods for more details), so as to produce a very small impedance (<1 Ω; on state) in the frequency range from DC to tens of kHz and thus, a very small voltage drops for forward biasing. These inductors also generate a very high impedance (>1000 Ohm; off state) at 64 MHz.

**Figure 7:**
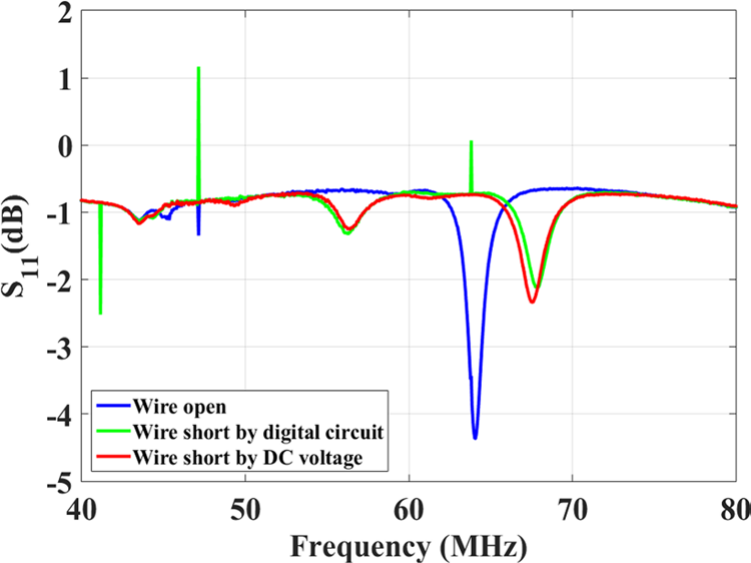
Measured resonance frequency of the metamaterial resonator when the switch matrix is “off” (blue line) and when the switch matrix turns “on” directly by a DC voltage (red line) and a clock signal of a digital circuit with a pickup inductor (green line).

**Figure 8:**
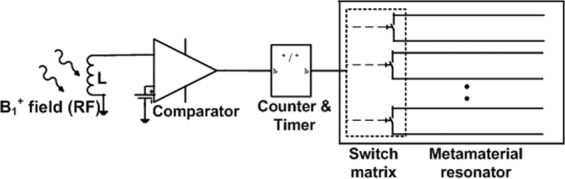
Schematic of the automatic switching mechanism. A pickup inductor generates a small AC signal induced by the B_1_^+^ field. The small AC (RF) signal is compared to a fixed DC voltage and digitized by a comparator which is down converted with the desired duty cycle by a counter and timer. The digitized signal is fed to an array of diode switches, each connected to a pair of parallel wires of the metamaterial resonator.

We carried out the experimental process in two stages. First, we used a DC source to activate and de-activate the diode switches directly and the resulting frequency shift is shown by the blue and red lines in Fig. 7. We then performed a second set of measurements using a digital electronic circuit with a pickup inductor (sensing magnetic field) that automatically changes the on/off states of the diode switches by creating a clock signal. In this test setup the pulsed magnetic field (simulating the RF pulses produced during the MRI sequence) originated from a small custom-build solenoid. Figure 8 shows the schematic of the automatic switching mechanism where the pickup inductor is used to sense the B_1_ field and generates a pulse to activate the switching mechanism. The pickup inductor could be used to activate the diodes directly, however due to their small size the voltage (current) induced via the B_1_ field might be too weak to switch the diode array. Therefore, we used a digital circuit for sensing the B_1_ field and converting it to a rail-to-rail set of pulses.

Figure 7 shows the frequency response of the metamaterial when the diode switches were turned on (green line) and off (blue line) by changing the RF signal amplitude applied to the solenoid (see Methods section). When the RF excitation turns the switches on, the wires are shorted, and the resonance frequency of the metamaterial splits into two peaks at 56.0 MHz and at 68.0 MHz. The 1.0 MHz difference in the resonance shift between the electronic and the DIP switching setups (Fig. 6(b)) is attributed to the absence of water at the switching end of the resonator, and the presence of inductors between the switches. This detuning via the splitting of the resonance is consistent with the detuning behaviour of conventional coils reported in the literature^30,31^. Other than the resonance of the metamaterial structure itself, there were few spikes (notably at 64 MHz) observed in the S_11_ spectrum which might arise due to the interaction of the loop with the solenoid fundamental and sub harmonics frequency spectrum.

### Evaluation of the switching mechanism in MRI for auto tuning

The final test was to evaluate the setup in a 1.5 T MRI scanner (Philips Ingenia, Best, The Netherlands) with a phantom mimicking the average human body conductivity and permittivity, placed on top of the resonator (details in methods). We used the body coil both as a transmit and receive coil during the scan. Initially, two scans were taken with the same low tip angle gradient echo sequence: a) with the metamaterial resonator detuned (wires shorted) for both the transmit (Tx) and receive (Rx) phases (a battery was used to bias and directly short the diodes); and (b) with the resonator tuned only during the receive phase, by using the pickup inductors and the digital circuit to switch automatically via the transmitted RF pulses.

**Figure 9:**
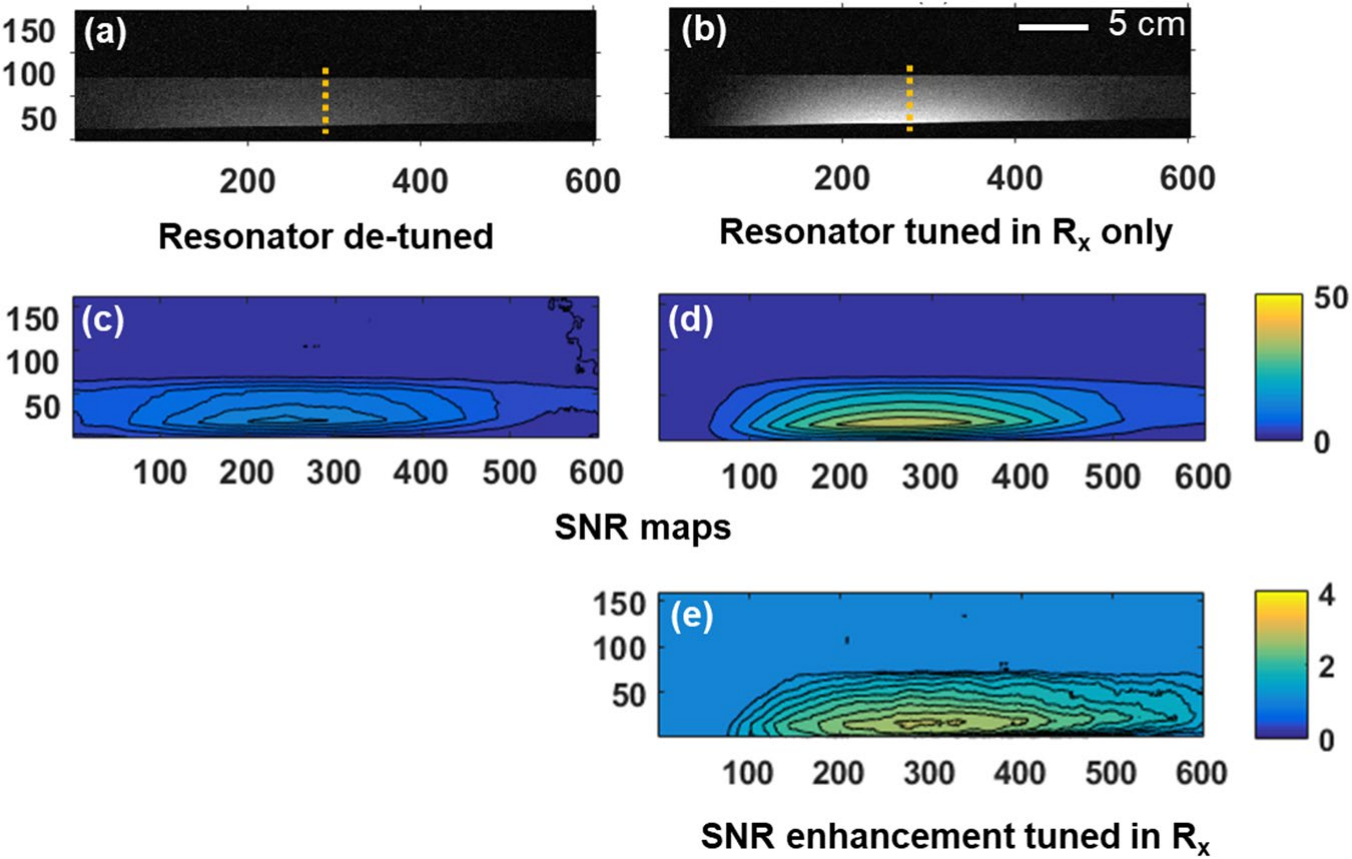
Pixel intensity and SNR maps for MRI scans. Pixel intensity for two scan setups when the metamaterial resonator is (**a**) detuned in both transmit and receive mode and (**b**) tuned only in receive mode using the auto tuning setup of the switching mechanism. (**c**,**d**) The respective SNR maps for the two scan setups. (**e**) SNR enhancement for the auto-tuned metamaterial (with the switch matrix) compare to the constantly detuned device. The colour bar represents the pixel intensity and SNR maps. The horizontal and vertical axes indicate pixel number.

**Figure 10:**
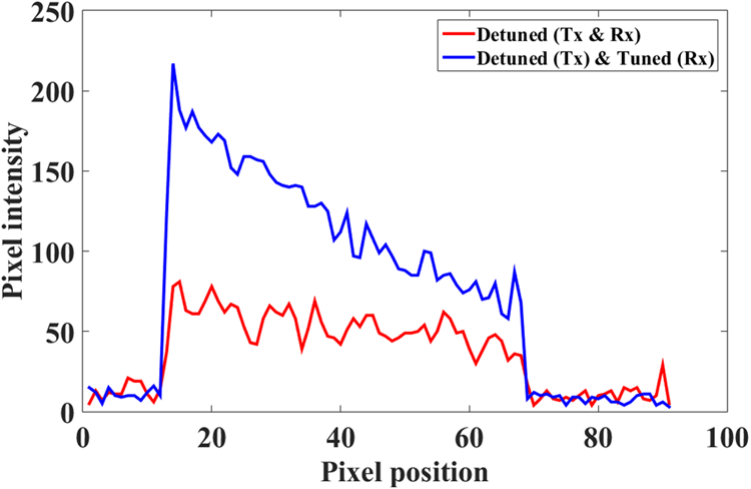
The one-dimensional map of the signal intensity through the centre of the phantom (orange line) from bottom to top. A significant increase in the pixel intensity was observed with the metamaterial. It is also observed that intensity reduce with distance from the resonator, which resemble any surface coil characteristic without intensity correction.

The two corresponding images (cases a & b) of the phantom along with the respective SNR maps in the region of interest are shown in Fig. 9. 1D projections through the middle of the phantom (dotted orange line, Fig. 9) are also plotted in Fig. 10. The transmit power was the same for the two cases, meaning that the local receive field is enhanced for the case resonator is tuned only in the receive sequence, which generates an image with improved signal intensity (Figs. 9b and 10) compared to that in Fig. 9a.

The same conclusions are drawn when observing the respective SNR maps (Fig. 9c,d). Figure 9e show the SNR enhancement when the metamaterial is Rx-tuned relative to when the metamaterial is detuned, as it graphically expresses the ratio $\frac{SN{R}_{Rx-tuned}}{SN{R}_{detuned}}$ . The maximum SNR improvement with the Rx-tuned metamaterial is 250%. These results validate that the switch matrix operates effectively in an MRI scanner. The signal intensity (Fig. 10) is reduced as a function of distance from the metamaterial, identical to the receive sensitivity of a loop coil^32^, since no intensity correction algorithm is applied. The improvement in SNR for this detunable metamaterial (Rx) is similar to the results obtained from an RF-sensitive non-linear metamaterial reported earlier^26^. However, our switching system is in principle agnostic to the metamaterial structure utilized for enhancement and could be adapted to control other types of resonators. The decrease in receive sensitivity away from the centre of the metamaterials is also consistent with earlier work^19,26^.

Finally, to validate that the switch matrix turns off the metamaterial resonator in the transmit sequence only, one additional scan was performed with the metamaterial resonator fully tuned (wires open) for both transmit and receive phases. The results are shown in Supplementary Data Figs. S1 and S2, showing further enhancement in SNR. However, as explained in the introduction, in practice it is more beneficial to tune the resonator in the receive phase only, as this additional enhancement in the transmit phase results in nonuniform field distribution.

In conclusion, we have presented a switching system for automatically detuning a wire-based metamaterial resonator during MRI scanning. The resonator is a passive device that locally enhances the receive magnetic field for improving the SNR in the region of interest. We first verified the detuning principle using manual slider switches. Subsequently, we used diodes and digital circuits for electronic switching and validated the mechanism on the bench using RF pulses generated through a solenoid. As a final test, we presented images from 1.5 T scans of tissue-mimicking phantoms which show that, due to the RF-sensitive switching system, the resonator detunes automatically during the transmit phase and tunes back to the Larmor frequency in the receive phase. No physical intervention or connection to the MRI console was required. By detuning the device in the transmit phase only, scanning can be performed safely using scanner-provided SAR models, the SNR is enhanced, and the transmit field homogeneity is preserved.

We will concentrate our future efforts on improving the uniformity of the B_1_ field enhancement profile by modifying the resonator design. Additionally, we aim to replace DI water with a high permittivity solid or semi-solid material to reduce the size of the metamaterial and to enable it to be incorporated into multi-element arrays.

### Consent to use image in the publication

Authors confirm that informed consent was obtained from the participants in the MRI measurements for using their image.

## Supplementary information

Supplementary information.
