# Supplementary material for: A smart switching system to enable automatic tuning and detuning of metamaterial resonators in MRI scans
Source: Sci Rep. 2020 Jun 22;10:10042. doi: 10.1038/s41598-020-66884-z (PMC7308377; doi:10.1038/s41598-020-66884-z)
Supplement: Supplementary file 1 — Supplementary information. [file 41598_2020_66884_MOESM1_ESM.docx]

**Supplementary Materials**:

**Title: A smart switching system to enable automatic tuning and detuning of metamaterial resonators in MRI scans**

Authors: Shimul Saha^1,2*^, Roberto Pricci^1,2^, Maria Koutsoupidou^1,3^, Helena Cano-Garcia^1,2^, Ditjon Katana^1^, Srinivas Rana^3^, Panagiotis Kosmas^1,2,3^, George Palikaras^1,2^, Andrew Webb^4^, Efthymios Kallos^1,2^

**Affiliations**:

1. MediWiSe| Medical Wireless Sensing Ltd, Queen Mary Bio Enterprise Innovation Centre, 42 New Road, E1 2AX, London, UK
2. Metamaterial Inc, 1 Research Drive, Dartmouth, Nova Scotia, B2Y 4M9, Canada
3. Department of Engineering, King’s College London, London, WC2R 2LS, UK
4. C.J. Gorter High Field Magnetic Resonance Center, Leiden University Medical Center, Albinusdreef 2, 2333 ZA Leiden, The Netherlands

The pixel intensity and SMR maps for fully tuned (Rx+Tx), detuned, and Rx-tuned scans (Fig. S1). The 1d pixel intensity for respective scan through the centre of the water phantom (Fig. S2).


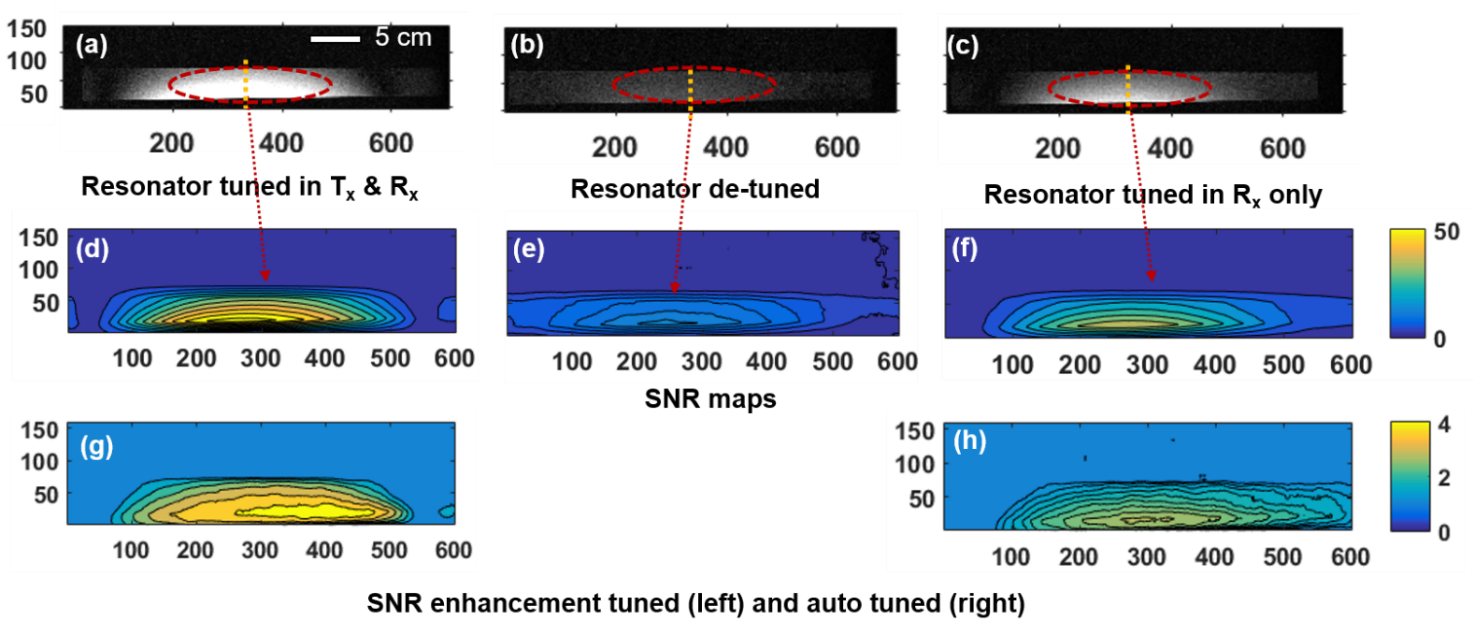


Figure S1. Pixel intensity and SNR maps. Pixel intensity for three scan setups: when the metamaterial resonator is (a) tuned in transmit and receive mode (b) detuned in transmit and receive mode and (c) tuned only in receive mode using the auto tuning setup of the switching mechanism. (d, e, f) The respective SNR maps for the three scan setups. SNR enhancement for (g) the constantly tuned metamaterial and for (h) the auto-tuned metamaterial (with the switch matrix) compare to the constantly detuned device.


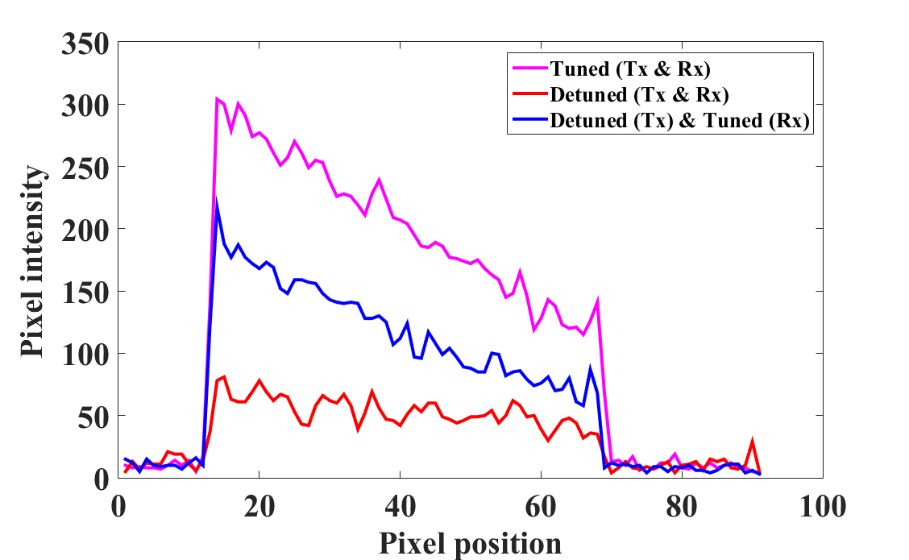


Figure S2. The one-dimensional map of the signal intensity through the centre of the phantom (orange line) from bottom to top. A significant increase in the pixel intensity was observed with the metamaterial. It is also observed that intensity reduce with distance from the resonator, which resemble any surface coil characteristic without intensity correction.
